# Intraoperative Assessment of Endogenous Microbiota in the Breast

**DOI:** 10.1055/s-0041-1736300

**Published:** 2021-11-16

**Authors:** Henrique Stachon, Vanessa Amoroso, Cicero Urban, Pamela Bioni, Cleverton Spautz, Rubens Silveira de Lima, Karina Anselmi, Flávia Kuroda, Iris Rabinovich, Thabata Alvarez, Juliane Monteiro

**Affiliations:** 1 Postgraduate Program, Biotechnology, Universidade Positivo, Curitiba, PR, Brazil; 2 Breast Unit, Hospital Nossa Senhora das Graças, Curitiba, PR, Brazil; 3 Microbiology Laboratory, Hospital Nossa Senhora das Graças, Curitiba, PR, Brazil

**Keywords:** surgery infection, capsular contracture, nipple-sparing mastectomy, breast conservative surgery, biofilm, infecção cirúrgica, contratura capsular, mastectomia com preservação do mamilo, cirurgia conservadora da mama, biofilme

## Abstract

**Objective:** Breast surgery is considered a clean surgery; however, the rates of infection range between 3 and 15%. The objective of the present study was to intraoperatively investigate the presence of autochthonous microbiota in the breast.

**Methods:** Pieces of breast tissue collected from 49 patients who underwent elective breast surgery (reconstructive, diagnostic, or oncologic) were cultured. The pieces of breast tissue were approximately 1 cm in diameter and were removed from the retroareolar area, medial quadrant, and lateral quadrant. Each piece of tissue was incubated in brain heart infusion (BHI) broth for 7 days at 37°C, and in cases in which the medium became turbid due to microorganism growth, the samples were placed in Petri dishes for culturing and isolating strains and for identifying species using an automated counter.

**Results:** Microorganism growth was observed in the samples of 10 of the 49 patients (20.4%) and in 11 of the 218 pieces of tissue (5%). The detected species were *Staphylococcus lugdunensis, Staphylococcus hominis, Staphylococcus epidermidis, Sphingomonas paucimobilis* , and *Aeromonas salmonicida.* No patient with positive samples had clinical infection postoperatively.

**Conclusion:** The presence of these bacteria in breast tissue in approximately 20% of the patients in this series suggests that breast surgery should be considered a potential source of contamination that may have implications for adverse reactions to breast implants and should be studied in the near future for their oncological implications in breast implant-associated large-cell lymphoma etiology.

## Resumo

**Objetivo** : A cirurgia de mama é considerada uma cirurgia limpa; entretanto, as taxas de infecção variam entre 3 e 15%. O objetivo deste estudo foi investigar no intraoperatório a presença de microbiota autóctone na mama.

**Métodos** : Pedaços de tecido mamário coletados de 49 pacientes submetidas à cirurgia eletiva da mama (reconstrutiva, diagnóstica ou oncológica) foram cultivados. Os pedaços de tecido mamário tinham aproximadamente 1 cm de diâmetro e foram removidos da área retroareolar e dos quadrantes medial e lateral. Cada pedaço de tecido foi incubado em caldo BHI ( *brain heart infusion* ) por 7 dias a 37 ° C, e nos casos em que o meio ficou turvo devido ao crescimento de microrganismos, as amostras foram colocadas em placas de Petri para cultivo e isolamento de cepas e para identificação de espécies usando um contador automatizado.

**Resultados** : O crescimento do microrganismo foi observado nas amostras de 10 das 49 pacientes (20,4%) e em 11 dos 218 pedaços de tecido (5%). As espécies detectadas foram *Staphylococcus lugdunensis* , *Staphylococcus hominis* , *Staphylococcus epidermidis* , *Sphingomonas paucimobilis* e *Aeromonas salmonicida* . Nenhum paciente com amostras positivas apresentou infecção clínica no pós-operatório.

**Conclusão** : A presença dessas bactérias no tecido mamário em aproximadamente 20% das pacientes desta série sugere que a cirurgia mamária deve ser considerada uma fonte potencial de contaminação que pode ter implicações nas reações adversas aos implantes mamários e deve ser estudada em um futuro próximo por suas implicações oncológicas na etiologia do linfoma de células grandes associado ao implante de mama.

## Introduction

Elective breast surgery, mastoplasty, and partial and total mastectomies are traditionally considered clean surgeries, according to their potential for contamination. 1 However, the rate of infection in patients who undergo elective breast surgery ranges between 3 and 15%, 2 which is above that expected for a surgery that is considered as clean. In patients with risk factors for infection, such as smoking, neoadjuvant chemotherapy, and previous radiotherapy, the infection rate may be as high as 25%. 3 The use of prophylactic antibiotics has reduced the infection rate from 15 to 9%, but this percentage is still above that expected for a surgery that is considered a clean one. 4

In addition to postoperative clinical infections, another common but late complication related to infection or to the presence of bacteria in the surgical area is capsular contracture. 5 This may occur in up to 30% of patients undergoing plastic or reconstructive surgery of the breast with implant placement. 6 The causes of capsular contracture are yet to be clarified, but the presence of subclinical infection is one of the involved factors. 7 One hypothesis is that the presence of bacteria in the surgical area from the breast ducts and parenchyma causes contamination of the implant, with the formation of a biofilm that is resistant to antibiotics. 8 The formation of a biofilm leads to chronic inflammation, which causes capsular contracture. In addition, this chronic inflammation around the implant covered by biofilm may lead to the development of breast implant-associated large-cell lymphoma. 9

The objective of the present study was to determine the presence of endogenous microbiota in the breast tissue intraoperatively, at different locations in the breast. The presence of endogenous bacteria in the breast tissue can explain why infection rates are higher than expected in breast surgery.

## Methods

### Patients

The present prospective study was approved by the internal review board (IRP) of Universidade Positivo. The study subjects were female patients undergoing elective breast surgery (mastoplasty and partial and total mastectomies with immediate breast reconstruction) at the Breast Unit of Hospital Nossa Senhora das Graças (Curitiba, PR); all patients were operated on by the same surgical team. The patients received information about the study and signed an informed consent form agreeing to participate in it. Forty-nine patients were recruited, with a total of 78 breasts (29 bilateral and 20 unilateral surgeries). Tissue samples were collected from each operated breast of the study patients. The pieces of tissue were representative of the retroareolar or central areas, medial , and lateral (axillary) quadrants, with the aim of comparing bacterial growth among the various samples in different positions of the breast.

In addition, the influence of the following clinical variables on the results of the cultures was investigated: diabetes mellitus, neoadjuvant chemotherapy, radiotherapy, menopause, breastfeeding, body mass index (BMI), purpose of the surgery (oncologic, cosmetic, or reconstructive), and presence of malignancy.

### Sample Collection

All the patients received prophylactic antibiotics preoperatively (cefazolin 1 g, intravenously during anesthesia induction). The skin was thoroughly prepared with 2% antimicrobial chlorhexidine (antisepsis) and with an alcohol-based solution of 0.5% chlorhexidine (asepsis) and subsequently covered with sterile surgical drapes, exposing only the area of the skin involved in the surgery. Intraoperative samples were collected from each breast in the first 30 min of surgery, from 3 different locations: retroareolar tissue (region 1), medial gland tissue (region 2), and axillary gland tissue (region 3). The samples consisted of pieces of tissue of at least 1 cm each, excised by a cold scalpel. The samples were placed in sterile flasks with saline solution (also sterile), stored in a cold room (4°C), and sent for culture in the microbiology laboratory of Universidade Positivo.

The samples were incubated in brain heart infusion (BHI) broth at 37°C in a shaker incubator, with constant shaking (150 RPM), for 7 days, and then they were subsequently assessed for turbidity. The samples that became turbid due to microorganism growth were inoculated in Petri dishes and placed in an incubator at 37°C for 7 days. Each turbid broth was used to inoculate two dishes using different techniques: the pour-plate technique for facultative/microaerophilic anaerobes, and the spread-plate technique for aerobes. Different strains were isolated from each dish and were inoculated in new dishes, with each strain placed in one quadrant of the dish. The cultures were sent to a private clinical analysis laboratory to identify the species using a Vitek 2 automated counter.

### Statistical Analysis

Data analysis started with the evaluation of the normality condition of the quantitative variables using the D'Agostino-Pearson test. Subsequently, the Student t-test was used for the quantitative variables that passed the normality test and Fisher exact test was used for the qualitative variables to detect differences between patients with positive and negative culture results (Zar, 2009). 10 The statistical analyses were performed using the GRAPHPAD PRISM statistical package, and the level of significance was set at 5% (α = 0.05).

## Results

The patients' ages ranged from 33 to 74 years (mean, 49 years; standard deviation, 8.48). Twenty-eight patients (57%) underwent oncologic surgery, 10 (20%) underwent diagnostic surgery, 8 (16%) underwent delayed reconstructive surgery, 2 (4%) underwent risk-reduction surgery, and 1 (2%) underwent one-stage oncologic surgery and risk-reduction surgery (oncologic surgery in one breast and risk-reduction surgery in the contralateral breast) ( Table 1 ).

**Table 1:** Type of surgery performed

Type of surgery	Frequency
Oncologic surgery	28 (57.1)
Diagnostic surgery	10 (20.4)
Reconstructive surgery	8 (16.3)
Risk-reduction surgery	2 (4)
Oncologic + risk-reduction contralateral surgery	1 (2)
Total	49 (100)

A total of 218 pieces of breast tissue were removed and processed from 78 breasts of 49 patients. Two patients had tumors in both breasts, 21 patients had a tumor in the right breast, and 23 patients had a tumor in the left breast. Regarding neoadjuvant therapies, 12 patients underwent chemotherapy before surgery and 6 underwent radiotherapy before surgery. Three patients were diabetic, 17 were menopausal, and 31 had breastfed. The patients' BMI varied between 18 kg/m ^2^ and 36 kg/m ^2^ (mean, 24.8 kg/m ^2^ ; standard deviation, 5.66).

The culture media of 13 samples from 12 different patients exhibited turbidity (positive result). Of these, two samples from two different patients did not exhibit bacterial growth in the laboratory. Ten of the 49 patients (20.4%) exhibited bacterial growth in at least one of the cultures of the sampled pieces. Eleven of the 218 samples (5%) presented cultures with bacterial growth. The identified bacteria were *Sphingomonas paucimobilis* (three patients), *Staphylococcus hominis* (one patient), *Staphylococcus lugdunensis* (three patients), *Staphylococcus epidermidis* (two patients, one of them had the microorganism in two different samples), and *Aeromonas salmonicida* (one patient) ( Table 2 ). No patient with positive samples for bacterial growth had clinical infection in the postoperative period. There were no significant differences between patients with and without a positive culture result with regard to the nine assessed variables ( Table 3 ).

**Table 2:** Identified species

Species	Frequency (n = 10)
*Staphylococcus lugdunensis*	30.0%
*Sphingomonas paucimobilis*	30.0%
*Staphylococcus epidermidis*	20.0%
*Staphylococcus hominis*	10.0%
*Aeromonas salmonicida*	10.0%

**Table 3:** Relationship between the clinical findings and purpose of surgery and the presence of endogenous microbiota in the breast

Variable analyzed		Positive (n = 10)	Negative (n = 38)	*P* -value
Age (years)		50.9 ± 9.2	48.6 ± 11.1	0.694
Purpose of surgery	Reconstruction	20.0%	15.8%	> 0.999
	Diag + RR	0.0%	2.6%	
	Oncologic	80.0%	81.6%	
Tumor location	Right	45.9%	44.4%	0.716
	Left	51.4%	44.4%	
	Bilateral	2.7%	11.1%	
DM (yes)		10.0%	5.4%	0.521
NC (yes)		10.0%	28.9%	0.414
RTX (yes)		20.0%	10.5%	0.591
BMI (kg/m ^2^ )		24.5 ± 1.6	24.9 ± 4.7	0.684
Menopause (yes)		44.4%	36.1%	0.711
Breastfeeding (yes)		70.0%	64.9%	> 0.999

Abbreviations: BMI, body mass index; Diag + RR, diagnostic and risk-reduction surgeries; DM, diabetes mellitus; NC, neoadjuvant chemotherapy; RTX, radiotherapy.

Among the samples with bacterial growth, four were from retroareolar tissue (three in the left breast and one in the right breast), four were from axillary tissue (one left axillary, one right axillary plus right medial, and two right axillary), and three were from medial tissue (one right medial, one left medial, and one right medial plus right axillary) ( Table 4 ).

**Table 4:** Locations of the positive cultures

Location of the positive culture	Frequency (n)
Retroareolar	4
Lateral	4
Medial	3

## Discussion

Conceptually, clean surgeries are those performed in tissues that are sterile or susceptible to decontamination, in the absence of a local infectious and inflammatory process or gross technical errors, elective and traumatic surgeries with first-intention wound healing and without drainage, and surgeries wherein there is no entry into the digestive, respiratory, or urinary tracts. Breast surgeries are traditionally categorized as clean surgeries. However, studies have reported higher rates of breast surgery infection than expected for this category. 2 3 The routine use of prophylactic antibiotics has significantly reduced the rates of infectious complications, but values that are higher than expected in clean operations are still found. In a meta-analysis involving 2,395 patients, Zhang (2014) 11 concluded that the use of prophylactic antibiotics reduces the rates of infection and prevents the development of other surgical complications, such as dehiscence, ischemia, and necrosis.

Capsular contracture is a frequent cause of reoperations, and its etiology is still unclear or lacking in consensus; however, it has been strongly associated with the presence of bacteria in the surgical area, 5 which form a film over an implant, known as biofilm. 12 A biofilm is defined as an adhesion layer between bacterial cells and an extracellular matrix, which is resistant to most antibiotics 5 13 14 and to physical and chemical methods of sterilization, because it blocks the penetration of gases and liquids. 15 The presence of biofilms is confirmed by sonication and implant culture, even in patients without signs of clinical infection. 16 Bacterial contamination is the factor with the greatest impact on capsular contracture formation, regardless of the type of implant lining. 17 Bacterial contamination leads to the formation of a thicker capsule with greater tissue reaction and a higher amount of inflammatory cells. 17 18

Irrigation of the surgical area with antimicrobial substances reduces the risk of developing capsular contracture, 19 another fact that supports the hypothesis that the presence of microbiota is a factor for the development of capsular contracture, as reported by Yalanis et al. (2015) 19 in a meta-analysis with 5,153 patients. Another late complication of mastoplasty with implants, anaplastic large-cell lymphoma, has been associated with the presence of biofilms. Breast implant-associated anaplastic large-cell lymphoma is a rare T-cell lymphoma that may develop around breast implants in plastic or reconstructive surgeries. 18 20 Chronic inflammation around the implant is thought to be the cause of lymphoma development, 18 20 21 and the presence of a biofilm around the implant is believed to promote inflammatory reactions, which increase the probability of developing lymphoma. 22 Removal of the capsule is the primary treatment for this type of lymphoma. 18 However, the presence of the bacterial component alone does not appear to be sufficient for the inflammatory stimulus required for the development of lymphoma; the surface component of the silicone implant is also needed. 23 Therefore, Santanelli di Pompeo (2015) 24 suggests that the only safe treatment to avoid breast implant-associated lymphoma is autologous flap breast reconstruction instead of implants.

Moreover, the presence of microbiota in nipple aspirate fluid has been demonstrated through the amplification and sequencing of nucleic acids. 25 Chan (2016) 25 compared the microbiota of nipple aspirate fluid of patients with a history of breast cancer with that of control patients and found abundant bacterial populations in both groups. Similarly, different surgical techniques are associated with different complication rates, with techniques involving incisions near the nipples and implants having higher rates. 26 27 28 The use of a funnel-shaped device that assists in implant placing to avoid contact between the implant and tissues during its positioning also reduces the rates of reoperation due to capsular contracture. 29 These facts may be explained by the presence of bacteria inside the breast ducts and by the intraoperative contamination of the implant by these bacteria. 7 A similar study with cultures of tissue samples collected from 50 breasts reported bacterial growth in 19 of them, resulting in 38% of breasts with cultures positive for microorganisms. 7 The authors of the study concluded that the breast harbors endogenous microbiota that may be the source of spontaneous or postoperative infections.

In the present study, *Sphingomonas paucimobilis* , a gram-negative bacillus, was found in 30% of the positive cultures. This microorganism is also present in the soil, plants, and potable water. It has been isolated from distilled water tanks, respirators, and hemodialysis equipment in hospital settings. Patients with chronic diseases or immunosuppression may be susceptible to infections by this microorganism. Hospital and community infections have been described, including sepsis, septic pulmonary embolism, peritonitis, septic arthritis, and endophthalmitis. 30 *Staphylococcus lugdunensis* was also detected in 30% of the positive cultures in the present study. It was first described in 1988 and was attributed an important role because of its capacity to cause serious infections, such as endocarditis; intra-abdominal infections; as well as infections of the skin and soft tissues, the central nervous system, and bones and joints. Its penicillin-resistance rate can be as high as 76%, varying between community and nosocomial strains. 31 *Staphylococcus epidermidis* , a typical gram-positive commensal microorganism of the human skin microbiota, was isolated in 20% of the positive cultures. It is a facultative anaerobe that is resistant to various environmental conditions. 7 Its pathogenic capacity is closely related to the capacity of biofilm formation of its strains, which makes it resistant to various hostile environments. 32 *Staphylococcus hominis* was present in 10% of the positive cultures. It is another gram-positive microorganism commonly present in the human skin, in animals of the human biome, and in the environment. It is a causative agent of infections in immunocompromised individuals. It is capable of facultative fermentation, as demonstrated by the isolation of lactate fermentation genes. 33 Finally, *Aeromonas salmonicida* was also isolated in 10% of the positive cultures. The genus comprises gram-negative, oxidase-positive, facultative anaerobes that are rod-shaped and widely distributed in the aquatic environment. It was considered a pathogen in cold-blooded animals only but has increasingly been reported as an opportunistic pathogen in humans, causing mainly gastrointestinal infections, furunculosis, and septicemia. 34

## Conclusion

Despite the preoperative use of prophylactic antibiotics, rigorous skin antisepsis, and adequate sterile surgical techniques, 20.4% of the patients in the present study had positive cultures. This number, although in a limited sample, is similar to that found in the literature, and the majority of the isolated species, which were staphylococci, were the same as those detected in other studies. Some of the detected species are associated with infections in immunocompromised patients. The locations with a higher number of positive cultures were the retroareolar area and the lateral quadrant, which is in line with the findings of other studies (not statistically significant). Thus, the breast harbors endogenous microbiota that may be responsible for the formation of biofilms and the contamination of implants and may even be associated with the pathophysiology of implant-associated large-cell lymphoma. Further studies are necessary to prove this hypothesis.
